# Comparison of Physicochemical Characteristics and Fibril Formation Ability of Collagens Extracted from the Skin of Farmed River Puffer (*Takifugu obscurus*) and Tiger Puffer (*Takifugu rubripes*)

**DOI:** 10.3390/md17080462

**Published:** 2019-08-07

**Authors:** Shan-Shan Wang, Ying Yu, Yong Sun, Nan Liu, De-Qing Zhou

**Affiliations:** 1 Yellow Sea Fisheries Research Institute, Chinese Academy of Fishery Sciences, Qingdao 266071, China; 2 Laboratory for Marine Drugs and Bioproducts, Pilot National Laboratory for Marine Science and Technology (Qingdao), Qingdao 266237, China

**Keywords:** puffer fish, acid-soluble collagen (ASC), pepsin-soluble collagen (PSC), fibril formation, fibril morphologies

## Abstract

Acid-soluble collagen (ASC) and pepsin-soluble collagen (PSC) from the skin of river puffer (ASC-RP and PSC-RP) and tiger puffer (ASC-TP and PSC-TP) were extracted and physicochemically examined. Denaturation temperature (T_d_) for all the collagens was found to be 25.5–29.5 °C, which was lower than that of calf skin collagen (35.9 °C). Electrophoretic patterns indicated all four samples were type I collagen with molecular form of (α_1_)_2_α_2_. FTIR spectra confirmed the extracted collagens had a triple-helical structure, and that the degree of hydrogen bonding in ASC was higher than PSC. All the extracted collagens could aggregate into fibrils with D-periodicity. The fibril formation rate of ASC-RP and PSC-RP was slightly higher than ASC-TP and PSC-TP. Turbidity analysis revealed an increase in fibril formation rate when adding a low concentration of NaCl (less than 300 mM). The fibril formation ability was suppressed with further increasing of NaCl concentration, as illustrated by a reduction in the turbidity and formation degree. SEM analysis confirmed the well-formed interwoven structure of collagen fibrils after 24 h of incubation. Summarizing the experimental results suggested that the extracted collagens from the skin of river puffer and tiger puffer could be considered a viable substitute to mammalian-derived collagens for further use in biomaterial applications.

## 1. Introduction

Puffer has been widely regarded as top cuisine in East Asia for their palatable taste and abundant nutrition. In particular, river puffer (*Takifugu obscurus*) and tiger puffer (*Takifugu rubripes*) are the two most common edible puffer species in China. However, the consumption of puffer had been limited in China for 26 years due to the potential threat of tetrodotoxin (TTX). TTX in wild puffer is accumulated mainly in the liver and ovary, through the food chain, starting from bacteria [1,2]. It has been reported that farmed puffer becomes nontoxic when fed with nontoxic diets in a cultivation environment where TTX-bearing organisms have been eliminated [3]. With the success of modern breeding technology, the production of farmed puffer is growing rapidly. In China, the production of puffer was approximately 22,993 tons in 2016, most of which was exported to Japan, South Korea and other Southeast Asian countries [4,5,6,7].

In 2016, the prohibition on consumption of farmed river puffer and tiger puffer was lifted by the Ministry of Agriculture and China Food and Drug Administration. Puffer aquaculture is now a thriving industry in China, and it is predicted that there will be a significant increase in puffer production in the next decade. Puffer skin is very thick and covered with tiny spines on the dorsal and ventral regions, which could be due to the fact that puffer inflate their anterior body to repel predators when a threatening situation arises [8]. However, thickness and small spines lead to a rough taste and make the skin undesirable among consumers. Therefore, it is expected that future food processing developments will isolate value-added biopolymers such as collagen from puffer skin in order to reduce the wasting of resources.

Collagen is the predominant structural protein found in animal connective tissues, including skin, bone, tendon, corneas and other load-bearing tissues [9]. Until now, at least 29 different types of collagen (type I-XXIX) consisting of polypeptide chains with distinct amino acid sequences have been identified. Collagen-based biopolymers have been ubiquitously applied in food and pharmaceutical fields, including food-packaging films, tissue scaffolds, cell culture systems, drug delivery vectors and artificial skins [10,11,12]. It has been reported that fibrillar collagen molecules are capable of spontaneously forming well-organized fibrils in vitro under ideal conditions via hydrophobic, electrostatic and hydrogen bonding interactions [13,14]. Fibril formation improves the specific surface area, enhances the resistance to enzyme degradation and strengthens the thermal stability of collagen-based materials [15]. Researchers have also demonstrated that collagen networks with nativelike fibrils exhibited better cellular response due to its analogous nature to extracellular matrix (ECM). Therefore, it is necessary to develop biomimetic collagen materials with nativelike fibrils for tissue engineering purposes [16,17].

Traditionally, the main sources of industrial collagen are the skin or tendon of bovine and porcine. However, because of the prevalence of zoonosis including bovine spongiform encephalopathy (BSE), foot-and-mouth disease (FMD), transmissible spongiform encephalopathy (TSE) and religious restrictions, the demand for collagen from alternative sources, especially from aquatic origin, has been increasing in recent years [18]. At present, aquatic collagens have been extracted and characterized from various species. Vertebrate sources include Nile tilapia, channel catfish, small-spotted catshark and golden pompano [19,20,21,22]. Invertebrate sources include jellyfish, squid and sponge [18,23,24]. However, previous reports have confirmed that collagens from different raw materials may have varying structures and characteristics, and exhibit very different fibril formation abilities, which have limited their wide-spread use. Fish collagen has a similar amino acid composition as conventional collagens with a decreased amount of imino acid and increased serine and threonine, and carries no disease risk [25]. To the best of our knowledge, there have been few reports comparing the characteristics of collagen from different puffer species. Thus, the aim of this study is to isolate and describe the physicochemical characteristics and in vitro fibril formation ability of collagens from the skin of farmed river puffer and tiger puffer in order to explore the possibility for applications in biomedical fields. In addition, the degree of fibril formation with different NaCl concentration has been investigated.

## 2. Results and Discussion

### 2.1. Amino Acid Composition and Thermal Denaturation Temperature (T_d_)

The amino acid composition of four collagen samples were expressed as amino acid residues per 1000 total amino acid residues (residues/1000 residues) and shown in Table 1. All four samples demonstrated the characteristic amino acid composition of collagen, with glycine (Gly) as the most abundant amino acid, followed by alanine (Ala), proline (Pro), glutamic acid (Glu) and arginine (Arg). The high Gly content is consistent with the understanding that the collagen molecule is featured with typical tripeptide repetitions (Gly-X-Y), except for the telopeptide regions (first 10 amino acid residues at the C-terminus and the last 14 of the N-terminus) [26].

In addition, the imino acid (Pro + Hyp) contents of collagens extracted from the skin of river puffer and tiger puffer were 196–199 and 190–192 residues/1000 residues respectively, which were similar to those of Bester sturgeon skin collagen (PSC: 192 residues/1000 residues) and giant croaker skin collagen (ASC: 194 residues/1000 residues, PSC: 191 residues/1000 residues) [27,28], whilst significantly higher than that of deep-sea redfish skin collagen (ASC: 165 residues/1000 residues) and Pacific cod skin collagen (ASC: 157 residues/1000 residues, PSC: 159 residues/1000 residues) [29,30]. However, the imino acid content was lower than collagens from striped catfish skin (ASC: 206 residues/1000 residues, PSC: 211 residues/1000 residues), calf skin (215 residues/1000 residues) and porcine skin (220 residues/1000 residues) [26,31,32]. The pyrrolidine rings of imino acids has been confirmed to play an important role in the structural integrity of triple helix by imposing restrictions on secondary structure changes of the polypeptide chains. Moreover, hydroxyproline contributes to the molecular stability by forming intramolecular hydrogen bond. Thus, higher imino acid content could lead to better thermal stability [33].

**Figure 1:**
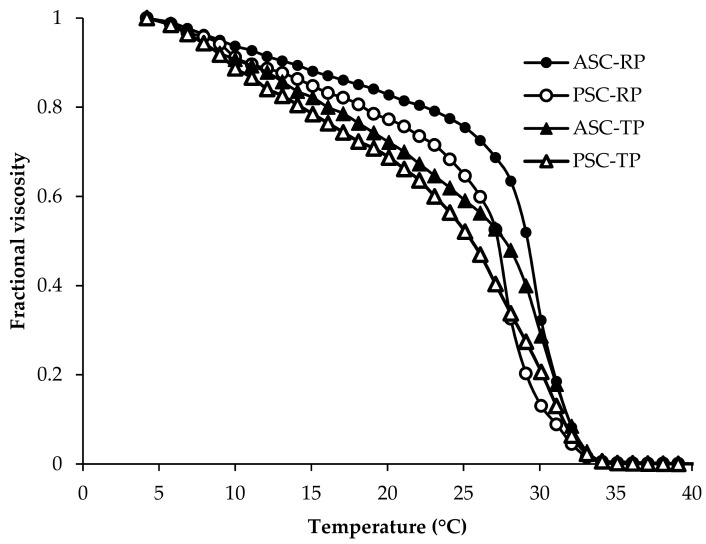
Thermal denaturation curves of collagens from the skin of river puffer (ASC-RP and PSC-RP) and tiger puffer (ASC-TP and PSC-TP), as shown by changes in fractional viscosity.

As shown in Figure 1, the denaturation temperature (T_d_) of ASC-RP, PSC-RP, ASC-TP and PSC-TP were 29.5, 27.5, 28.0 and 25.5 °C respectively, which were higher than cold-water species such as deep-sea redfish (16.1 °C) and Pacific cod (14.5–16 °C) [29,30]. Meanwhile, the T_d_ values of collagens extracted from river puffer and tiger puffer skins were lower than those of calf skin (35.9 °C) [34]. This result was consistent with the amino acid composition mentioned above. T_d_ values of pepsin-soluble collagens in this study were slightly lower than those of corresponding acid-soluble collagens, which might be caused by the molecular weight reduction due to the pepsin hydrolysis, especially in the telopeptide region [35]. Low thermal stability could lead to inferior mechanical strength of collagen-based materials, which limit their use in particular tissues. Although T_d_ values of river puffer and tiger puffer skin collagen are lower than those of land-based mammals, they are higher than those of cold water species, which is an advantage for application in the biomedical materials field [25].

### 2.2. Sodium Dodecyl Sulfate Polyacrylamide Gel Electrophoresis (SDS-PAGE)

**Figure 2:**
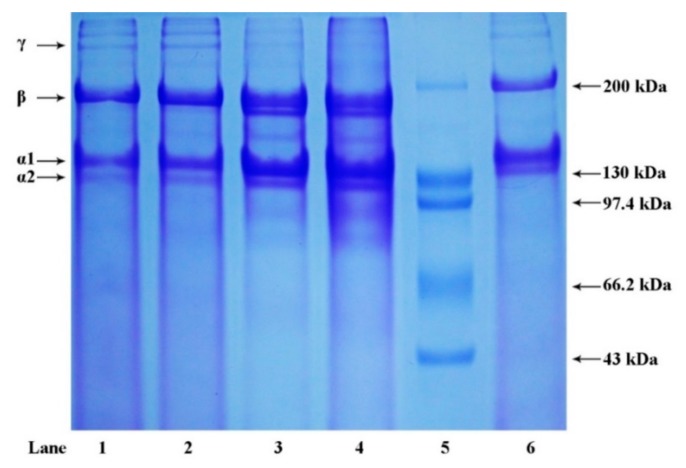
SDS-PAGE patterns of collagens from the skin of river puffer (ASC-RP and PSC-RP) and tiger puffer (ASC-TP and PSC-TP). Lane 1: ASC-RP; lane 2: PSC-RP; lane 3: ASC-TP; lane 4: PSC-TP; lane 5: protein marker; lane 6: bovine type I collagen.

Protein patterns of extracted collagens determined by SDS-PAGE are shown in Figure 2. Collagens from the skin of river puffer and tiger puffer exhibited a similar pattern, mainly consisting of at least two α-chains (α1 and α2), β-chains (dimers) and small amounts of γ-chains (trimers). The band intensity ratio of α1-chain to α2-chain was approximately 2:1, which indicated that the major collagens in river puffer and tiger puffer skins were most likely to be type I collagen, a heterotrimer containing two identical α1-chains and one α2-chain in the molecular form of (α1)_2_α2 [9]. This finding was similar to previous reports of skin collagens from other fish species [25,26,27,28]. Moreover, the amount of γ-chains in ASC-RP and PSC-RP was higher than that of ASC-TP and PSC-TP, suggesting that collagen extracted from river puffer skin contained more intermolecular and intramolecular cross-links. This result was also consistent with the better thermal stability of ASC-RP and PSC-RP mentioned above.

### 2.3. Fourier Transform Infrared Spectroscopy (FTIR)

**Figure 3:**
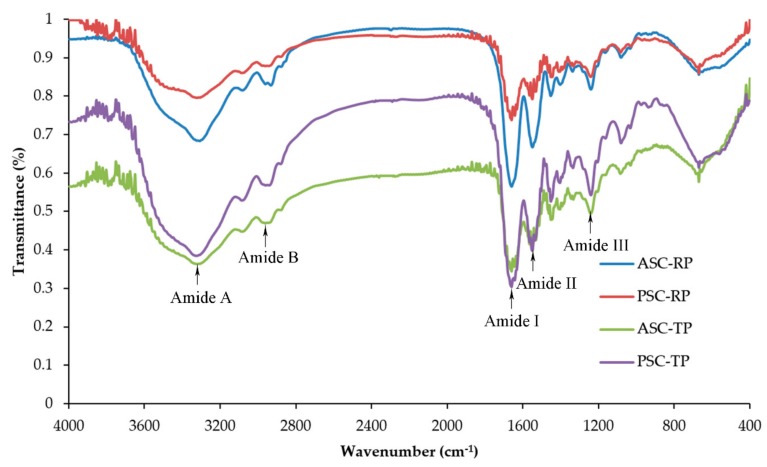
FTIR spectra of collagens from the skin of river puffer (ASC-RP and PSC-RP) and tiger puffer (ASC-TP and PSC-TP).

The major absorption bands in the FTIR spectra are shown in Figure 3. Amide A is associated with the N–H stretching frequency which occurs in the range of 3400 cm^−1^ to 3440 cm^−1^. With the N–H groups involved in the formation of hydrogen bonds, the wavenumber shifts to a lower frequency [36]. The Amide A bands of ASC-RP, PSC-RP, ASC-TP and PSC-TP were measured at 3307.79, 3321.29, 3319.37 and 3327.08 cm^−1^, respectively. This result suggested that the number of hydrogen bonds in acid-soluble collagens was higher than the number of bonds in the corresponding pepsin-soluble collagens. The peaks at 2931.69–2943.26 cm^−1^ were denoted as Amide B band associating with asymmetrical stretching vibrations of CH_2_ [37].

Amide I, II and III bands are related to the degree of molecular order and therefore involved with the triple-helical structure of collagen. Amid I band, which is mainly associated with the C=O stretching vibrations and hydrogen bonds coupled with COO^−^, usually occurs within the range of 1600–1700 cm^−1^ [38]. Amid II band is responsible for the N–H bending coupled with C-N stretching vibrations, with a characteristic absorption peak near 1550 cm^−1^. Amide III band generally appears at 1200–360 cm^−1^, and arises from the C–N stretching, N–H bending vibration as well as the wagging vibration of CH_2_ groups of the glycine backbone and the proline side-chains [19]. As shown in Figure 3, there were no obvious differences in Amide I (1656.79–1658.72 cm^−1^), Amide II (1548.78–1550.71 cm^−1^) and Amide III peaks (1238.25–1240.18 cm^−1^) among ASC-RP, PSC-RP, ASC-TP and PSC-TP, which indicated a high extent of helical structure maintained in extracted collagens. Moreover, absorption bands around 1390–1455 cm^−1^ were also found, which extensively corresponded to pyrrolidine ring vibration of hydroxyproline and proline. The absorption ratio between Amide III and 1452.34 cm^−1^ of all four samples was approximately 1.0, further confirming the presence of a native helical structure [39].

### 2.4. Fibril Formation Ability

**Figure 4:**
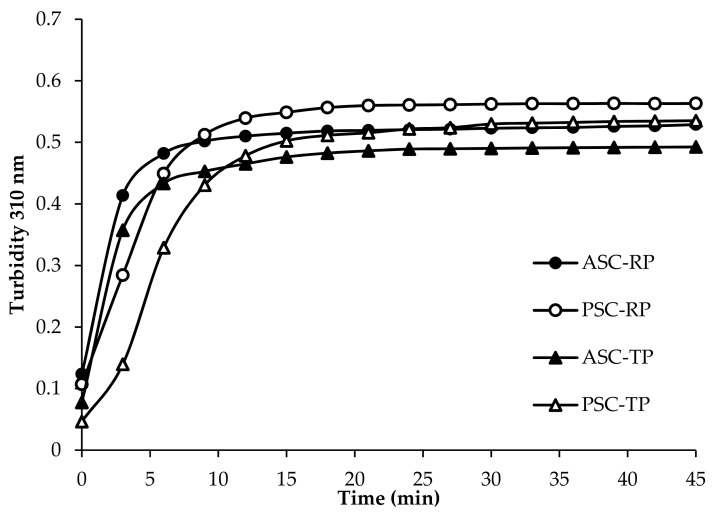
Fibril formation curves of collagens from the skin of river puffer (ASC-RP and PSC-RP) and tiger puffer (ASC-TP and PSC-TP). Fibril formation was monitored by the increase in turbidity at 310 nm.

Turbidity is a simple and effective method to investigate the mechanism and kinetics of fibril formation in solution. During formation process, turbidity changes from a nearly transparent solution into an opaque suspension full of fibrils that scatters large amounts of light [40]. In this study, the fibril formation of extracted collagens during an incubation at 25 °C was monitored by turbidity changes at 310 nm. As shown in Figure 4, there was no lag time in all four samples, suggesting a high speed of fibril nucleation. The river puffer skin collagens exhibited a higher rate of turbidity increase compared with tiger puffer skin collagens. Similar curves were found in collagens extracted from tilapia, Bester sturgeon and grass carp skin [27,41,42]. Although the ultimate turbidity was somewhat higher, the fibril formation speed of pepsin-soluble collagens was slightly lower than their corresponding acid-soluble collagens, which suggested that the nucleation time might be prolonged for the hydrolysis of pepsin. According to the literature report, telopeptide sequences are important in stabilizing the initial nucleation. Enzymatic hydrolysis of the telopeptide regions prior to fibril formation may suppress the fibril formation process [43,44,45].

**Figure 5:**
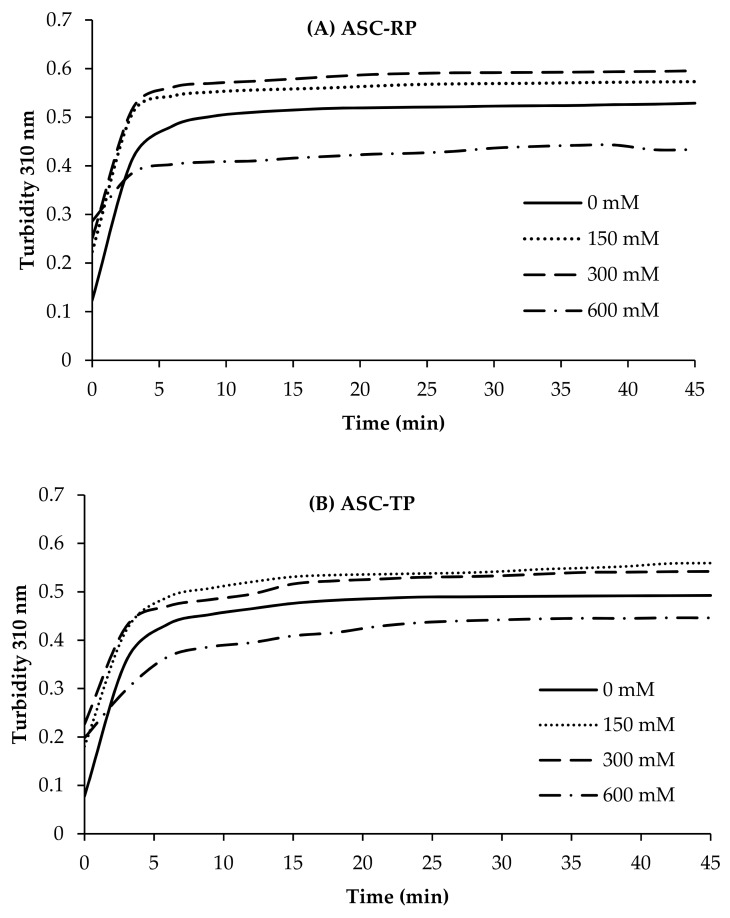
Effect of NaCl concentration on the fibril formation ability of collagens from the skin of river puffer (**A**) (ASC-RP) and tiger puffer (**B**) (ASC-TP).

Figure 5 shows the fibril formation curves of ASC-RP and ASC-TP solutions with increasing ionic strength by the addition of NaCl. The fibril formation speed of ASC-RP and ASC-TP was obviously sped up with the addition of NaCl (0–300 mM). The increase in fibril formation speed demonstrated an acceleration effect of low concentration of NaCl. Fibril formation curves of PSC-RP and PSC-TP with different concentrations of NaCl exhibited similar tendencies (Data not shown). This result was consistent with the previous report of collagen from Bester sturgeon skin [27]. A possible explanation is that there are many positively charged (e.g., lysine and arginine) or negatively charged (e.g., aspartic and glutamic acid) functional side-chains of amino acid residues on the surface of the collagen triple-helical structure. At low ionic strength, the addition might bind with these groups on the collagen surface, which would renormalize the effective charge distribution of the triple-helical surface, thereby reducing the electrostatic repulsion and enhancing the fibril formation process [46]. However, with the continuing increase of NaCl concentration, the fibril formation ability of both collagens began to decrease, suggesting that high ionic strength could suppress the fibril formation process. The collagens from barramundi and tilapia skin were also inhibited by high NaCl concentrations [47]. It is reported that the addition of higher concentrations of inorganic ions would increase the net charge in the triple-helical surface, leading to greater electrostatic repulsion. Therefore, the collagen aggregates in the solution tended to disperse as the NaCl concentrations continued to increase [48,49].

**Figure 6:**
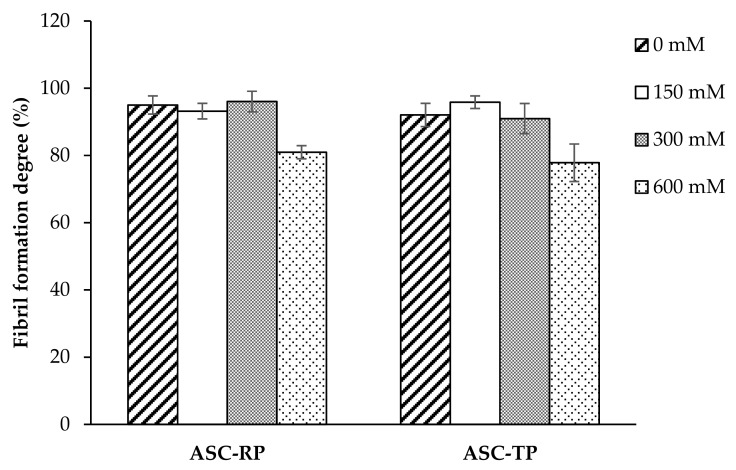
Effect of NaCl concentration on fibril formation degree of collagens from the skin of river puffer (ASC-RP) and tiger puffer (ASC-TP). Error bars show standard deviation.

When comparing the fibril formation degree (Figure 6), it could be found that both ASC-RP and ASC-TP showed degree values higher than 90% without NaCl addition. This result was similar to collagen from Bester sturgeon skin [27], while higher than those of collagens from grass carp skin (28.0%) [42], catla and rohu skin (36–78%) [50]. Furthermore, the fibril formation degree of ASC-RP and ASC-TP remained high (>90%) with NaCl concentration ranging from 150 to 300 mM. However, at the high NaCl concentration of 600 mM, the degree values of both samples had decreased to 80.95% and 77.82%, which was consistent with the turbidity measurement result (Figure 5). Collagen monomers, which failed to aggregate into fibrils with the increasing NaCl concentration, present as random and clustered accumulation of collagen assemblies without nucleation [46]. In contrast to this result, the fibril formation degree of salmon skin collagen and jellyfish collagen continuously decreased with the increasing NaCl concentration [51,52]. The conflicting degree values might be explained in part by the species differences and the experimental conditions under which fibril formation is initiated [53]. Overall, the present study demonstrated that collagen from river puffer and tiger puffer skin had an impressive fibril formation ability under the positive effect of NaCl, but the extent of its influence varied greatly depending on the concentration. For the industrial use of puffer skin collagen in biomedical fields, it will be necessary to further optimize the fibril formation conditions.

### 2.5. Transmission Electron Micrographs (TEM) of Collagen Fibrils

**Figure 7:**
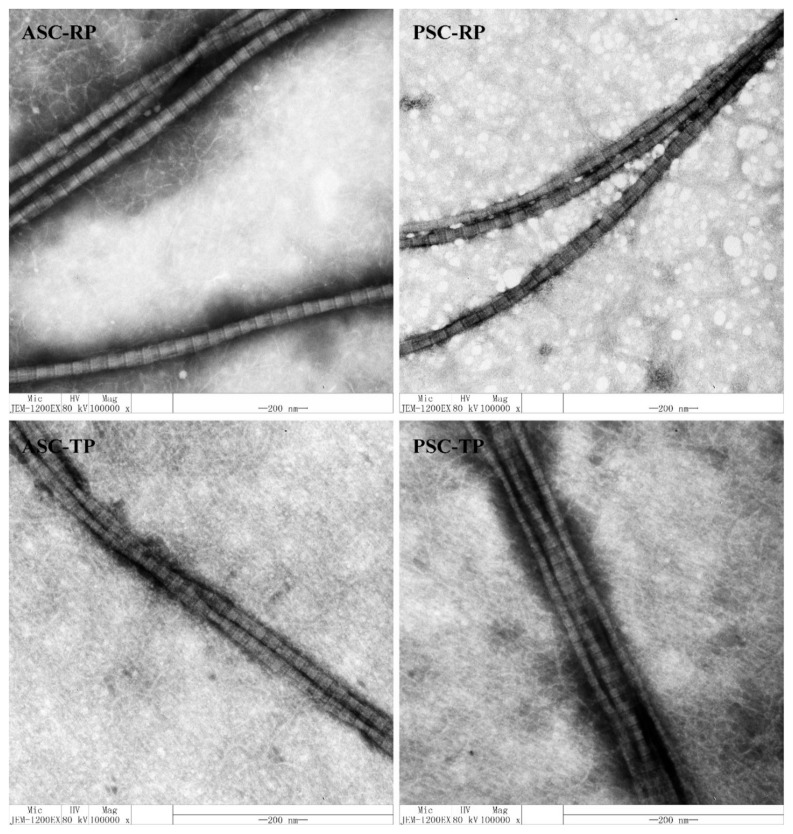
TEM observations of fibrils formed by collagens from the skin of river puffer (ASC-RP and PSC-RP) and tiger puffer (ASC-TP and PSC-TP).

TEM images of fibrils from river puffer and tiger puffer are illustrated in Figure 7. All four samples could aggregated into fibrils with a periodic cross-striped structure (D-periodicity). The average D-periodicity of ASC-RP (68.83 ± 2.79 nm), PSC-RP (69.20 ± 2.57 nm), ASC-TP (68.77 ± 2.48 nm) and PSC-TP (68.05 ± 1.50 nm) was similar to collagen isolated from porcine skin (approximately 67 nm) [54]. Previous studies have shown that intrafibrillar mineralization occurs only when the collagen fibrils are well organized with native D-periodicity, this organization results in the proper alignment of the positively charged amino acids of neighboring molecules into domains that, in turn, are able to interact with amorphous calcium phosphate and mediate its infiltration into the gap zones of fibrils [46,55]. Therefore, collagen extracted from the river puffer and tiger puffer holds great promise as suitable biomaterials for bone tissue engineering scaffolds.

### 2.6. Scanning Electron Micrographs (SEM) of Collagen Fibrils

**Figure 8:**
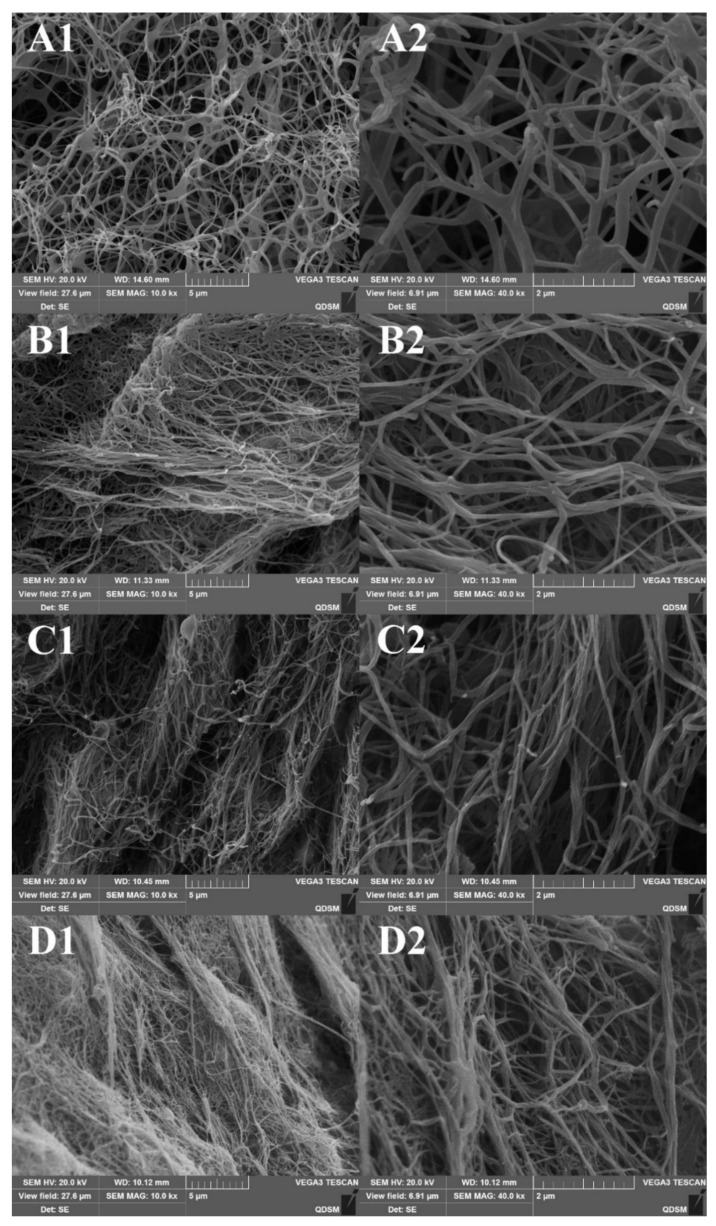

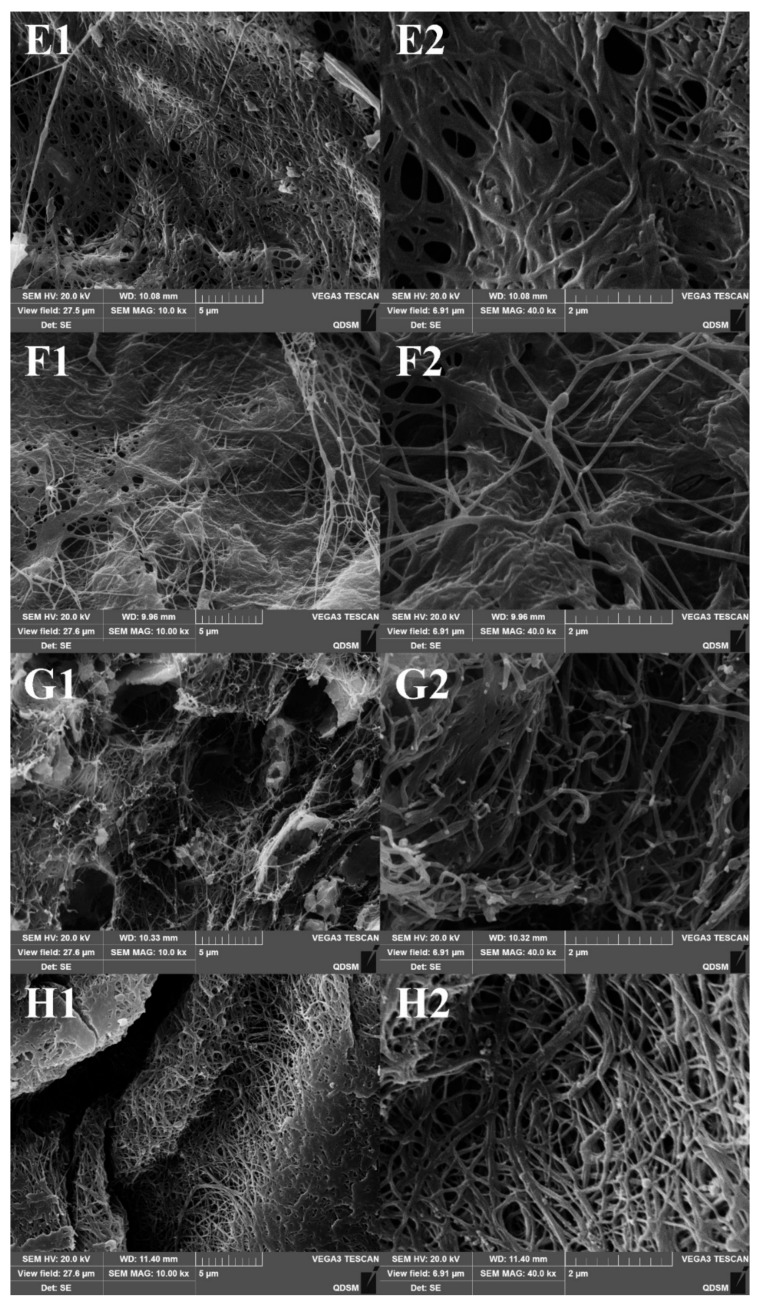
SEM observations of fibrils formed by collagens from the skin of river puffer (ASC-RP and PSC-RP) and tiger puffer (ASC-TP and PSC-TP). (**A**–**D**): ASC-RP, PSC-RP, ASC-TP and PSC-TP incubated for 1 h; (**E**–**H**): ASC-RP, PSC-RP, ASC-TP and PSC-TP incubated for 24 h.

The SEM morphologies of collagen fibrils after 1 h of incubation are shown in Figure 8A–D. All four samples showed a complex characterization. The diameter of resultant fibrils was approximately in the range of 70–140 nm. Thick fibril bundles with diameter larger than 200 nm were also observed, which was formed by the side-to-side association of fibril monomers. After 24 h of incubation, it was evident that the slender fibrils of all four samples fused into interwoven three-dimensional networks which were compact and well-formed (Figure 8E–H). This result indicated that the fusion of collagen fibrils needed longer time than fibril formation itself. The SEM ultrastructure of the resultant fibrils was similar with those of collagens from Egyptian Nile tilapia scale and Bester sturgeon skin [27,31]. It is widely recognized that the uniform and consistent reticular structure of fibrils serves as a significant favorable property for biomedical and pharmaceutical application of collagen [56]. These results suggested that fibril recombined collagen from the skin of rive puffer and tiger puffer have the potential to be an alternative source for valuable biopolymers. Further studies on the biocompatibility and immune response of these collagen-based biomaterials with human cells are needed for practical applications [57].

## 3. Materials and Methods

### 3.1. Materials and Chemicals

The skins of river puffer (*T. obscurus*) were obtained from Jiangsu Zhongyang Co., Ltd., in Nantong City, Jiangsu Province of China. The skins of tiger puffer (*T. rubripes*) were collected from Dalian Tianzheng Co., Ltd., in Dalian City, Liaoning Province of China. These skins were cleaned with cold distilled water and stored at −20 °C before being used. Porcine pepsin (500 U/mg, dry matter) was purchased from Beijing Biotopped Science and Technology Co., Ltd. (Beijing, China). High-molecular weight protein marker and bovine type I collagen were purchased from Solarbio Science and Technology Co., Ltd. (Beijing, China). Other reagents used were of analytical grade.

### 3.2. Extraction of Acid-Soluble Collagen (ASC)

All procedures were conducted at 4 °C to reduce chain fragmentation according to the method of Li et al. with a slight modification [35]. Thawed skins were soaked with 0.1 M NaOH at a solid to liquid ratio of 1:20 (*w*/*v*) for 12 h to remove non-collagenous protein and pigment, and then washed with pre-cooling distilled water until pH was neutral. The pretreated skins were homogenized and soaked with 0.5 M acetic acid in a ratio of 1:50 (*w*/*v*) for 48 h with continuously gentle stirring. The mixture was centrifuged (ST16R, Thermo Scientific, IL, USA) at 8500 r/min for 20 min. The residues were re-extracted for 48 h and the supernatants were combined. The precipitate was slowly salted-out by adding grinded NaCl to a final concentration of 0.9 M, and collected by centrifuging at 8500 r/min for 20 min. The precipitate was re-dissolved in a minimum volume of 0.5 M acetic acid. The resulting solution was dialyzed against 0.1 M acetic acid for 24 h and replaced with distilled water for 48 h. Both dialysates were renewed every 8 h. The collagen sample was freeze-dried (CoolSafe 55, ScanLaf A/S, Lynge, Denmark) and referred to as acid-soluble collagen from tiger puffer (ASC-TP) and acid-soluble collagen from river puffer (ASC-RP).

### 3.3. Extraction of Pepsin-Soluble Collagen (PSC)

The pretreatment procedures were similar to that of ASC. The fish skins after alkali treatment and homogenization were extracted with 0.5 M acetic acid (1:50 *w*/*v*) in the presence of 0.5% (*w/w*) pepsin. The mixture was continuously stirred for 48 h, followed by centrifugation and precipitation as described above. The resulting precipitate after centrifugation was re-dissolved in 0.5 M acetic acid and dialyzed against 0.02 M Na_2_HPO_4_ for 24 h to inactivate pepsin before being further dialyzed against distilled water. The obtained collagens were referred to as pepsin-soluble collagen from tiger puffer (PSC-TP) and pepsin-soluble collagen from river puffer (PSC-RP).

### 3.4. Amino Acid Composition

Tested samples were hydrolyzed under reduced pressure in 6 M HCl at 110 °C, and then vaporized. The hydrolysates were dissolved in 25 mL citric acid buffer and applied to an amino acid analyzer (Hitachi 835-50, Hitachi High-Technology Corporation, Tokyo, Japan). The hydroxyproline (Hyp) content was determined according to the chloramine T method described by Sun et al. [14].

### 3.5. Determination of Denaturation Temperature (T_d_)

The denaturation temperature (T_d_) was determined by measuring the viscosity change using a MCR101 viscometer (Anton Parr Co., Ltd., Shanghai, China) [58,59]. The lyophilized sample was dissolved in 0.1 M acetic acid to a concentration of 4 mg/mL. Fractional viscosity was calculated by the formula:

Fractional viscosity = (η_sp(measured)_ − η_sp(minimum)_)/(η_sp(maximum)_ − η_sp(minimum)_) (1)

where η_sp_ is the specific viscosity. T_d_ was determined as the temperature at which the change in fractional viscosity was 50% decreased.

### 3.6. SDS-PAGE

Electrophoretic patterns of collagens were determined using a previously published method with 7.5% running gel and 4% stacking gel [60]. The sample solutions were neutralized and centrifuged at 9200 g for 20 min. The resulting supernatants were mixed with loading buffer (60 mM Tris-HCl, pH 8.0, containing 25% glycerol, 2% SDS, 0.1% bromophenol blue) at a ratio of 4:1 (*v*/*v*) in the presence of β–ME, and electrophoresed in an electrophoresis system (DYCZ-25D, Liuyi Corporation, Beijing, China). A high molecular weight protein maker was used to estimate the molecular weight of the proteins. After electrophoresis, the gel was stained in Coomassie blue R-250 (0.1%, *w*/*v*) staining solution for 1 h and discolored overnight.

### 3.7. FTIR Spectral Analysis

FTIR spectra were measured from discs containing 2 mg of lyophilized sample mixed with 100 mg of dried spectrum pure potassium bromide (KBr). The spectra results were recorded using a FTIR spectrophotometer (Bruker Optik GmbH, Ettlingen, Germany) with a 2 cm^−1^ resolution in the 4000–400 cm^−1^ range.

### 3.8. Determination of Fibril Formation Ability

Fibril formation ability was measured according to a modified previously published method [27]. Samples were dissolved in 0.5 M acetic acid to 1 mg/mL concentration. The solution was mixed with equal volumes of 0.1 M Na-phosphate buffer (pH 7.2) with various NaCl concentrations (0, 150, 300 and 600 mM). The mixture was kept at 25 ± 1 °C. The fibril formation curve was observed by measuring the turbidity change of the mixture at 310 nm via a spectrophotometer (UV-2550, Shimadzu Co., Ltd., Kyoto, Japan). After the fibril formation experiment, the mixture was centrifuged at 8500 r/min for 15 min. Fibril formation degree was calculated as the percentage of the decrease of hydroxyproline content in the supernatant.

### 3.9. Transmission Electron Microscopy (TEM)

After the turbidimetric assay, one drop of fibril mixture was loaded on a 200-mesh copper grid and stained with 1% (*w*/*v*) phosphotungstic acid, according to the method of Yousefi [33]. TEM images of skin collagen fibrils were observed using a transmission electron microscopy (JSM-1200, JEOL Ltd., Tokyo, Japan).

### 3.10. Scanning Electron Microscopy (SEM)

The SEM observations of centrifugated fibrils were obtained by the method of Zhang et al. [27] with slight modifications. Fibril samples were fixed with 2.5% (*v*/*v*) glutaraldehyde in 0.1 M phosphate buffer (pH 7.2) for 3 h, and then rinsed with distilled water. Dehydration was performed in a graded series of ethanol concentration (70, 80, 90, 95 and 100%). Thereafter, fibrils were soaked in two 30 min changes of *t*-butyl alcohol solution, and then freeze-dried. The lyophilized samples were pasted on a blade with double side adhesive tape and then coated with gold using a fine coater (JFC-1200, JEOL Ltd., Tokyo, Japan). The ultrastructure was observed using a scanning electron microscope (JSM-840, JEOL Ltd., Tokyo, Japan).

## 4. Conclusions

River puffer and tiger puffer are the major puffer varieties produced in China. The processing of these puffer varieties generates large quantities of skin by-products. In this report, ASC and PSC from the skin of river puffer and tiger puffer were successfully extracted and characterized for their potential as biomaterials. The proline and hydroxyproline contents of ASC-RP, PSC-RP, ASC-TP and PSC-TP were 196, 199, 190 and 192 residues/1000 residues, respectively. Thermal stability of river puffer and tiger puffer skin collagen was found to be lower than calf skin collagen, while higher than cold-water fish species. SDS-PAGE profile and FTIR spectra indicated all four samples were undenatured type I collagen with two different α chains, the amount of γ chains in ASC-RP and PSC-RP was higher than that in ASC-TP and PSC-TP. All the collagens were capable of aggregating into fibrils in vitro. The fibril formation rate of river puffer skin collagen was slightly higher than the collagen from tiger puffer skin. Turbidity assay revealed that a low concentration of NaCl had a positive effect of promoting fibril formation. The striped D-periodicities of resultant fibrils could be observed via TEM, which could contribute to the mechanical stability and biological functions of collagen-based materials. SEM observation confirmed that all four collagens could form branched and interlaced slender fibrils, and the fibrils could further develop into a more compact reticular structure after 24 h of incubation. Overall, these results suggest that the extracted collagens from river puffer and tiger puffer skin could be utilized as alternatives to terrestrial collagens as a biomedical material, and may enhance the added value of this fish species.

## Figures and Tables

**Table 1:** Amino acid composition of collagens from the skin of river puffer (acid-soluble collagen river puffer (ASC-RP) and pepsin-soluble collagen river puffer (PSC-RP)) and tiger puffer (acid-soluble collagen tiger puffer (ASC-TP) and pepsin-soluble collagen tiger puffer (PSC-TP)) (residues/1000 residues).

Amino Acid	ASC-RP	PSC-RP	ASC-TP	PSC-TP	Calf Skin Collagen [31]	Porcine Skin Collagen [32]
Aspartic acid (Asp)	46	44	45	47	45	44
Threonine (Thr)	22	23	22	21	18	16
Serine (Ser)	39	38	40	38	33	33
Glutamic acid (Glu)	72	69	73	74	75	72
Glycine (Gly)	326	331	332	339	330	341
Alanine (Ala)	126	124	127	122	119	115
Cysteine (Cys)	2	1	2	1	0	0
Valine (Val)	28	28	27	24	21	22
Methionine (Met)	9	11	10	14	6	6
Isoleucine (Ile)	10	9	9	9	11	10
Leucine (Leu)	18	17	17	18	23	22
Tyrosine (Tyr)	4	3	3	2	3	1
Phenylalanine (Phe)	10	15	10	13	3	12
Histidine (His)	6	7	5	7	5	5
Lysine (Lys)	29	28	30	27	26	27
Arginine (Arg)	57	53	58	52	50	48
Proline (Pro)	116	116	114	115	121	123
Hydroxyproline (Hyp)	80	83	76	77	94	97
Imino acid (Pro + Hyp)	196	199	190	192	215	220
